# Linkage to HIV care, postpartum depression, and HIV-related stigma in newly diagnosed pregnant women living with HIV in Kenya: a longitudinal observational study

**DOI:** 10.1186/s12884-014-0400-4

**Published:** 2014-12-03

**Authors:** Bulent Turan, Kristi L Stringer, Maricianah Onono, Elizabeth A Bukusi, Sheri D Weiser, Craig R Cohen, Janet M Turan

**Affiliations:** Department of Psychology, University of Alabama at Birmingham, CH 415, 1530 3rd Avenue South, Birmingham, AL 35294-1170 USA; Department of Sociology, University of Alabama at Birmingham, HHB 460, 1720 2nd Ave South, Birmingham, AL 35294-1152 USA; Centre for Microbiology Research, Kenya Medical Research Institute (KEMRI), Nairobi, Kenya; Department of Medicine, University of California, San Francisco, San Francisco, CA USA; Department of Obstetrics, Gynecology and Reproductive Sciences, University of California, San Francisco, San Francisco, CA USA; Department of Health Care Organization and Policy, School of Public Health, University of Alabama at Birmingham, Birmingham, AL USA

**Keywords:** HIV, Stigma, Postpartum, Depression, Linkage to care

## Abstract

**Background:**

While studies have suggested that depression and HIV-related stigma may impede access to care, a growing body of literature also suggests that access to HIV care itself may help to decrease internalized HIV-related stigma and symptoms of depression in the general population of persons living with HIV. However, this has not been investigated in postpartum women living with HIV. Furthermore, linkage to care itself may have additional impacts on postpartum depression beyond the effects of antiretroviral therapy. We examined associations between linkage to HIV care, postpartum depression, and internalized stigma in a population with a high risk of depression: newly diagnosed HIV-positive pregnant women.

**Methods:**

In this prospective observational study, data were obtained from 135 HIV-positive women from eight antenatal clinics in the rural Nyanza Province of Kenya at their first antenatal visit (prior to testing HIV-positive for the first time) and subsequently at 6 weeks after giving birth.

**Results:**

At 6 weeks postpartum, women who had not linked to HIV care after testing positive at their first antenatal visit had higher levels of depression and internalized stigma, compared to women who had linked to care. Internalized stigma mediated the effect of linkage to care on depression. Furthermore, participants who had both linked to HIV care and initiated antiretroviral therapy reported the lowest levels of depressive symptoms.

**Conclusions:**

These results provide further support for current efforts to ensure that women who are newly diagnosed with HIV during pregnancy become linked to HIV care as early as possible, with important benefits for both physical and mental health.

## Background

Postpartum depression is an important health problem for many new mothers [1-3], and has negative physical and psychological consequences for infants as well [4-9]. The prevalence of postpartum depression is estimated to be between 10%-15% in developed countries [10], and similar or even higher rates have been reported for low- and middle-income countries, including countries in sub-Saharan Africa [11-18]. Many sub-Saharan countries also have high rates of HIV infection among pregnant and childbearing women [19]. Although there have been efforts to improve care for women living with HIV in sub-Saharan Africa, postpartum depression among HIV-positive mothers remains understudied.

Pregnant women living with HIV may experience numerous additional stressors, including financial hardships [20,21], reduced social support [22], and concern for the physical wellbeing of their children [23-25]. These additional stressors may increase the risk for postpartum depression symptoms among HIV-positive women [14,15,17,18]. Furthermore, women who discover their HIV-positive status during pregnancy may be more likely to develop depressive symptoms than those who were already aware of their HIV-positive status before pregnancy [24].

HIV-related stigma may be another important factor contributing to depressive symptoms for pregnant women living with HIV. Stigma and discrimination around HIV are common in sub-Saharan Africa, and have been associated with adverse health outcomes. Specifically, among pregnant women, HIV-related stigma has repeatedly been shown to be a major barrier to HIV testing and counseling [26], utilization of labor and delivery services [27], and participation in prevention of mother-to-child transmission (PMTCT) programs [26,28-30]. HIV-related stigma has also been found to be associated with depression in the general population of persons living with HIV [31-41]. However, there is very little research examining stigma and depression simultaneously among pregnant or postpartum women in developing countries. In one cross-sectional study in South Africa, researchers found that experienced stigma (having experienced actual acts of discrimination) and internalized stigma (when persons living with HIV internalize negative attitudes perceived to be associated with HIV, resulting in feelings of low self-worth) were strong predictors of postpartum depression among HIV-positive women [14]. Similarly in Thailand, researchers found that over 75% of HIV-positive postnatal women reported that they felt that HIV is a disease of which their family would be ashamed, and women who reported this familial shame were significantly more likely to report depressive symptoms and HIV-related worry during the postpartum period [23].

While depression and stigma may impede linkage to HIV care, as well as adherence to antiretroviral therapy (ART) [42], it is also possible that linkage to HIV care, including receipt of ART, impacts both HIV-related internalized stigma and depression. One potential mechanism for this effect may be that linkage to HIV care may change perceptions related to the implications of having HIV. It has been suggested that as perceived treatment efficacy increases for stigmatized illnesses, social stigma may become less intense [43]. This would likely apply to persons living with HIV as well: Individuals linked to HIV care may have more positive attitudes regarding their HIV, and experience less internalized stigma, which may lead to higher self-esteem and feelings of being in control, which in turn may predict lower depression symptoms. It is also possible that linkage to care leads to lower stigma and depression by improving the physical well-being of people living with HIV [44].

Research examining the relationship between linkage to care, stigma, and depression in the general population of persons living with HIV has mostly focused on one component of HIV care: access to ART. A 2009 systematic review of 21 publications (some using longitudinal designs around the time when ART was first made available) examining the impact of ART on various outcomes in developing countries revealed that individuals receiving ART experience less depressive symptoms than those not receiving ART, even after controlling for physical health improvements [45-49]. The association between ART and internalized stigma is less consistent. A few studies of stigma and ART in African countries have suggested that ART is associated with improved self-image and lower stigma among persons living with HIV [44,50-53]. The rollout of ART has had significant impact on health in Sub-Saharan Africa, and recent work has suggested that the ability to work and earn a living increases following ART initiation [54,55], resulting in lower HIV-related stigma [56]. However, other studies in sub-Saharan Africa have presented conflicting results about the extent to which ART predicts lower HIV-related stigma [57-60]. Furthermore, these relationships have not been examined in pregnant or postpartum women living with HIV.

It is important to note that linkage to comprehensive “HIV care and treatment” extends beyond access to ART medications, especially for pregnant women living with HIV. However, few studies have investigated linkage to HIV care itself in terms of impacts on mental health and internalized HIV stigma. Many HIV care facilities in resource-limited settings are designed to provide additional services such as prevention education, family planning counseling, infant feeding counseling, food by prescription, couples HIV counseling and testing, and the opportunity to participate in support groups. Understanding the unique roles of early linkage to care and ART initiation for pregnant women living with HIV has particular relevance as these countries move towards Option B+, a strategy which recommends early initiation of life-long ART for all pregnant and postpartum women testing HIV-positive [61]. Despite the push for option B+, currently in many countries only those pregnant women meeting certain eligibility criteria are offered ART.

In this study, we investigated the associations among linkage to HIV care, depression, and internalized stigma in a population highly vulnerable to stigma and depression: newly diagnosed HIV-positive pregnant women in rural sub-Saharan Africa (women who tested HIV-positive for the first time at their first antenatal care visit). We hypothesized that linkage to HIV care after testing HIV-positive during pregnancy would be related to lower odds of having depression (and lower depressive symptoms) and lower internalized stigma at six weeks postpartum. Furthermore, we expected that the effects of linkage to HIV care on depression symptoms would be partially explained by internalized stigma. Finally, we examined effects on postpartum depression symptoms for women linked to Pre-ART (linked to HIV care but not on ART yet) versus ART care.

## Methods

### The setting

The data were obtained as part of a larger prospective study—the Maternity in Migori and AIDS Stigma (MAMAS) Study in Southern Nyanza Province, Kenya [30]. The rate of HIV infection among MAMAS participants was 18%, which is very similar to the rate estimated by Kenyan Antenatal Care (ANC) Sentinel Surveillance for this region [30,62]. The study clinics were Kenyan Ministry of Health facilities supported by Family AIDS Care & Education Services (FACES), a PEPFAR-funded program run by a collaboration between the Kenya Medical Research Institute (KEMRI) and the University of California, San Francisco (UCSF). All of the clinics had on-site HIV care and treatment programs and provided antiretroviral drugs for PMTCT prophylaxis to all pregnant women testing HIV-positive. Although eligibility for highly active antiretroviral therapy (HAART) was restricted to women with more advanced HIV disease (CD4 count < 350 or WHO Stage III/IV), all HIV-positive pregnant women were encouraged to enroll in the on-site HIV care and treatment program, which provided access to health education, adherence counseling services, patient support groups, and other supportive services. Recruitment, baseline interviews, and HIV testing took place between November 2007 and April 2009.

### Participants

Participants in the larger MAMAS study included women recruited from nine antenatal care (ANC) clinics (N = 1777). However, one of the clinics did not provide any follow-up data and was therefore excluded from current analyses. All women attending a first ANC visit at the study clinics during the study period were approached, and if they met eligibility criteria, they were asked if they would like to participate in the study. Participants were at least 18 years old, in their first seven months of pregnancy, who were visiting the ANC clinic for the first time in their pregnancy, and who did not know their current HIV status. After providing signed informed consent, participants responded to interviewer-administered questionnaires immediately before their ANC clinic visit. Information on participants’ acceptance of HIV testing during the ANC visit, test results, and subsequent linkage to care was obtained from their medical records. The characteristics of the sample at baseline have been described elsewhere [63].

A total of 598 women with valid locator information were selected for participation in follow-up interviews approximately six weeks after the birth. All women who either tested positive or refused testing at their first antenatal visit were automatically selected for follow-up, as well as a random sample of women who tested negative (follow-up sample: 226 HIV-positive, 227 HIV-negative, and 145 HIV status unknown). Of the HIV-positive women selected for follow-up, 154 (68%) participated in a follow-up questionnaire approximately six weeks after birth. As reported elsewhere, no important differences were detected between those women who were interviewed postpartum and those lost-to-follow-up [63]. Our analysis focuses on HIV-positive women who participated in both baseline and postpartum follow-up interviews, and who had complete (non-missing) data on key variables (n = 135). Participants were on average 5 months pregnant at the time of the baseline interview, with limited variability (Mean = 5.0, SD = 1.0).

### Measures

All survey measures and interviews were in the participants’ preferred language (Dholuo, Kiswahili, or English).

#### Socio-demographics

Variables utilized in the current analyses included age, education, marital status, number of births, and ownership of a mobile phone as a proxy for wealth [27,64].

#### Internalized stigma

Internalized stigma was measured during the follow-up postpartum interview using the self-stigma subscale of HIV/AIDS Stigma Instrument–PLWA (HASI-P) [65], which contains five items asking participants to report how often they experience different stigma-related emotions. The HASI-P was developed and validated for use in African countries, and is available in several African languages. In the present study, the HASI-P self-stigma scale had good internal consistency (Cronbach’s α = .87).

#### Postpartum depression

At follow-up, during the postpartum quantitative interview, postpartum depression was measured using the Edinburgh Postpartum Depression Scale (EPDS) [66,67]. The EPDS is an internationally validated scale developed specifically to assess postpartum depression and is the most widely used measure for postpartum depression in low- and middle-income settings [33,68-70]. Because the EPDS does not include somatic symptoms of depression, it is unlikely that HIV-related symptoms overlap with EPDS measures of depression, and the EPDS has been validated for use in HIV-positive populations in Africa [16,18]. EPDS consists of 10 self-reported items. In the present study, the EPDS had good internal consistency (Cronbach’s α = .82). We used the total EPDS scale score as a continuous variable to reflect postpartum depression symptom severity, as well as a binary variable obtained by dichotomizing scores at the suggested cut-off score of 13 for diagnosing major depression [71].

#### Linkage to HIV care

We defined linkage to HIV care as enrolling in an HIV care and treatment program and having at least one HIV care visit. Clinic enrollment records were examined to determine whether or not each participant became linked with the HIV care and treatment program available in their community after the baseline interview and prior to the postpartum follow-up interview.

### Other predictors in the current analyses

#### Partner communication about birthplace

At the postpartum interview, participants were asked the following question: “Did you discuss where you would deliver the baby with your husband/male partner before the birth?” (Yes/No).

#### Infant Health status

At the postpartum interview, participants were asked “Has the baby had any serious health problems since the birth?” (Yes/No).

#### Family knows about HIV status

At the postpartum interview, each participant was asked whether or not their family members know about their HIV-positive status (Yes/No), thus including both intentional and unintentional disclosure.

#### Pre/Postpartum intimate partner violence (IPV)

IPV was measured both at late pregnancy and postpartum. Participants were asked whether or not they had experienced emotional, psychological, physical, or sexual abuse during pregnancy or after the birth. Participants who reported IPV at either time period were categorized as “yes”, those who chose not to answer for either period were categorized as “unknown”, and those who reported no IPV at both time periods were categorized as “no” [28].

#### ART use

During the postpartum interview, women were asked to report if they were currently taking any antiretroviral HIV medications (Yes/No).

### Statistical analysis

Generalized estimating equations (GEE) were used to examine the effect of linkage to HIV care on internalized stigma and on depression symptoms as continuous variables, taking into account clustering within the study sites. Analyses also controlled for socio-demographic characteristics and other variables associated with postpartum depression from previous research: age, marital status, education, mobile phone ownership (as a proxy for wealth [27,64]), intimate partner violence, infant’s health status, number of births, disclosure status, and partner communication about birth place [16,25,72]. We also examined the effect of linkage to HIV care on depression as a dichotomized diagnostic variable (EPDS ≥ 13). Next, we repeated the analysis examining the effect of linkage to HIV care on depressive symptoms, with internalized stigma added as a predictor variable to the equation. To examine whether internalized stigma mediated the effect of linkage to HIV care on depression symptoms, we conducted exploratory mediation analyses using the ml_mediation program, which utilizes the procedures that Krull and MacKinnon [73] proposed for calculating and testing mediation effects (i.e., indirect effects) in clustered data. Lastly, we conducted GEE analyses to compare postpartum depression symptoms for the following three groups: a) not linked to HIV care, b) linked to HIV care but not on ART, c) linked to HIV care and on ART.

### Ethical review

This study received ethical approval from the Kenya Medical Research Institute (KEMRI) Ethical Review Committee, the University of California, San Francisco Committee on Human Research, and the University of Alabama at Birmingham Institutional Review Board. All women provided written informed consent for participation in the questionnaires and abstraction of data from their medical records.

## Results

Descriptive statistics for the main study variables are presented in Table 1. Of the 135 women included in the analyses, 34 (25.2%) were depressed according the EPDS criteria of a score of ≥ 13. Fifty-six participants had not linked to HIV care, whereas 79 participants had linked to HIV care (58.5%). As expected, depression scores were significantly correlated with internalized stigma scores (*r* = .56, *p* < .01).

**Table 1:** **Characteristics of the sample (N = 135 HIV positive women)**

**Variable**	**Mean (SD) or percentage** ^**a**^
***Socio-demographic characteristics***	
Mean age	24.26 (4.87)
Marital status (%)	
Not currently married	12.6
Currently married	87.4
Educational level (%)	
Primary or less	88.1
More than primary	11.9
Household goods (%)	
Owns a mobile phone	41.5
Does not own a mobile phone	58.5
***HIV-related characteristics***	
Mean HASI-P Internalized HIV-related Stigma score	0.64 (0.81)
Linked to HIV care (%)	
Yes	58.5
No	41.5
Family knows about HIV status (%)	
No	82.2
Yes	17.8
***Pregnancy-related characteristics***	
Partner communication about birthplace (%)	
No discussion of delivery with partner before the birth	32.6
Discussion of delivery with partner	67.4
Pre/postpartum intimate partner violence (%)	
Yes	28.1
No	31.9
Unknown	40.0
Mean number of births	2.10 (1.61)
Infant health status (%)	
No serious health problems after birth	80.7
Serious health problems after birth	19.3
Mean postpartum depression score (EPDS)	7.94 (5.59)
EPDS category (%)	
EPDS ≥13 possible depression	25.2
EPDS <13	74.8

Note. ^a^Mean (SD) are presented for continuous variables.

### Linking to HIV care and postpartum depression

Non-linkage to HIV care was a significant predictor of postpartum depression symptoms: Women who had not linked to HIV care had higher levels of depression compared to women who had linked to HIV care (See Table 2 for statistical results). Other significant predictors of postpartum depressive symptoms included intimate partner violence (women experiencing partner violence and women who had missing data on violence had higher levels of depression), disclosure (women whose HIV status is known to someone in their family had higher levels of depression), number of births (women with fewer births had higher levels of depression), infant’s health problems (women whose babies had health problems had higher levels of depression), and partner communication about the birth place (women who did not discuss the location of the upcoming birth with their partner had higher levels of depression).

**Table 2:** **Multivariable generalized estimating equation analysis predicting postpartum depression severity (N = 135)**

***Variable***	***Unstandardized B (Std. error)***	***p-Value***
Age	.08 (0.14)	.562
Marital status (ref: not currently married)		
Currently married	−0.01 (0.94)	.996
Educational level (ref: more than primary)		
Primary or less	−1.82 (1.76)	.301
Mobile phone ownership (ref: yes)		
No	-.59 (1.05)	.576
Pre/postpartum intimate partner violence (ref: none)		
Yes	4.67 (0.84)	.000
Unknown	2.26 (0.77)	.003
Infant health problems after birth (ref: no)		
Yes	3.47 (0.72)	.000
Number of births	−0.66 (0.28)	.019
Family knows about HIV status (ref: no)		
Yes	1.35 (0.66)	.040
Spousal communication about birthplace (ref: no)		
Yes	−2.31 (0.61)	.000
Linked to care (ref: no)		
Yes	−1.85 (0.91)	.043

Next, we repeated the GEE analyses using the dichotomized depression variable (EPDS ≥ 13, using binary logistic models). Linkage to care was significantly associated with lower odds of depression (odds ratio (OR) = 0.33, 95% CI = 0.16 - 0.69). Thus, this analysis also indicated that women who had not linked to HIV care were more likely to have symptoms consistent with a diagnosis of postpartum depression compared to women who had linked to HIV care.

### Linkage to care and internalized stigma

Linkage to HIV care was also negatively associated with internalized stigma. Women who had not enrolled in care had higher levels of stigma at the time of the postpartum interview, compared to women who had enrolled, controlling for covariates (see Table 3 for statistical details). Other significant predictors of internalized stigma were intimate partner violence (women experiencing partner violence and women who had missing data on violence had higher levels of stigma), disclosure (women whose HIV status is known to someone in their family had higher levels of stigma), number of births (women with fewer births had higher levels of stigma), and partner communication about the birth place (women who did not discuss the location of the upcoming birth with their partner had higher levels of stigma).

**Table 3:** **Multivariable generalized estimating equation analysis predicting internalized HIV-related stigma (N = 135)**

***Variable***	***Unstandardized B (Std. error)***	***p-Value***
Age	0.02 (0.02)	.282
Marital status (ref: not currently married)		
Currently married	0.05 (0.21)	.818
Educational level (ref: more than primary)		
Primary or less	−0.43 (0.33)	.188
Mobile phone ownership (ref: yes)		
No	−0.12 (0.07)	.086
Pre/postpartum intimate partner violence (ref: none)		
Yes	0.35 (0.14)	.011
Unknown	0.26 (0.16)	.022
Infant health problems after birth (ref: no)		
Yes	0.15 (0.13)	.237
Number of births	−0.10 (0.05)	.037
Family knows about HIV status (ref: no)		
Yes	0.37 (0.19)	.046
Spousal communication about birthplace (ref: no)		
Yes	−0.25 (0.12)	.039
Linked to care (ref: no)		
Yes	−0.34 (0.15)	.024

### The mediating role of stigma

Next, we repeated the GEE analysis with symptoms of postpartum depression as the outcome and with internalized stigma added to the equation as a predictor (see Table 4 for statistical details). In this model, internalized stigma was a significant predictor of depression: Women with higher internalized stigma had higher levels of depression. With stigma in the model, linkage to HIV care was no longer a significant predictor of postpartum depression symptoms. Mediation analysis for clustered data revealed a significant indirect effect of linkage to HIV care on depression, mediated by stigma (coefficient = −.61, *p* < .05, 95% bias-corrected confidence interval = −1.22 to -.11). Thus, this analysis provided evidence that stigma mediated the effect of linkage to HIV care on lower depression symptoms.

**Table 4:** **Multivariable generalized estimating equation analysis predicting postpartum depression severity with internalized stigma added to the model (N = 135)**

***Variable***	***Unstandardized B (Std. error)***	***p-Value***
Age	0.03 (0.10)	.764
Marital status (ref: not currently married)		
Currently married	−0.15 (0.48)	.765
Educational level (ref: more than primary)		
Primary or less	−0.57 (0.85)	.502
Mobile phone ownership (ref: yes)		
No	−0.24 (0.92)	.792
Pre/postpartum intimate partner violence (ref: none)		
Yes	3.67 (0.87)	.000
Unknown	1.50 (0.51)	.004
Infant health problems after birth (ref: no)		
Yes	3.03 (0.83)	.000
Number of births	−0.38 (0.23)	.098
Family knows about HIV status (ref: no)		
Yes	0.28 (0.88)	.746
Discussion of delivery with partner (ref: yes)		
No	1.60 (0.43)	.000
Linked to care (ref: no)		
Yes	−0.86 (0.713)	.226
HASI-P internalized HIV-related stigma	2.88 (.570)	.000

Next, we repeated the GEE analyses using the dichotomized depression variable (EPDS ≥ 13, using binary logistic models) with internalized stigma added as a predictor. In this model, stigma was a significant predictor of depression status (OR = 3.67, 95% CI = 1.87 - 7.22): Women with higher internalized stigma were more likely to be depressed. With stigma in the model, linkage to care was still a significant predictor of depression status; however, the effect of linkage was reduced (OR = 0.45, 95% CI = 0.22 - 0.90).

### The role of ART

Of the 79 participants included in the GEE multivariate analyses who had linked to HIV care, 33 (42%) were on ART. We compared postpartum depression symptoms for the following three groups: a) not linked to HIV care, b) linked to HIV care but not on ART, c) linked to HIV care and on ART. GEE analyses with this three-category variable (and the control variables) suggested that both of the first two groups (not linked to HIV care and linked but not on ART) had significantly more postpartum depression symptoms than the third group (linked and on ART; B = 3.20 and B = 2.26, respectively, both p values <= 0.001). There were no significant differences between the first two groups (not linked to HIV care versus linked but not on ART; B = 0.94, *p* = 0.37). The estimated marginal means for postpartum depression symptoms for the three groups were: 11.95, 11.01, and 8.75, respectively for a) not linked to HIV care, b) linked but not on ART, and c) linked and on ART.

Binary logistic models using the dichotomized depression variable as the dependent variable (depressed defined as EPDS ≥ 13) led to similar results. Both of the first two groups (not linked to HIV care and linked but not on ART) were significantly more likely to be depressed than the third group (linked and on ART; OR = 7.91, 95% CI = 2.79 – 22.44 and OR = 4.33, 95% CI = 1.80 – 10.40, respectively, both p values <= 0.001). There were no significant differences between the first two groups (not linked to HIV care versus linked but not on ART; OR = .55, 95% CI = .25 – 1.18, *p* = 0.12).

## Discussion

We examined postpartum depression symptoms and internalized stigma levels six weeks after birth for women who tested HIV-positive for the first time during their first ANC visit. We found that HIV-positive pregnant women who had linked with HIV care had significantly lower levels of depression symptoms (as well as lower postpartum depression rates using the recommended cutoff score for major depression) at around six weeks after the birth, as compared to HIV-positive women who did not link with HIV care. We also identified an important potential mediator for this effect: It appears that linkage to HIV care is associated with lower internalized HIV-related stigma, which in turn predicts lower postpartum depression symptom severity. ART seemed to be an important component of this effect on depression symptoms, with depression levels being lowest in women who were both linked to HIV care and on ART. This last finding (although based on a small sample size) lends further support to current efforts to roll out Option B+, the WHO-recommended strategy of initiating all HIV-positive pregnant women on lifelong ART, regardless of initial stage of HIV disease [74].

The findings of the current study are particularly relevant as the global community struggles to achieve optimal maternal and child health outcomes in sub-Saharan Africa, including HIV-free infant survival. HIV-infected pregnant women in sub-Saharan Africa are a highly vulnerable group (especially those who become aware of their HIV infection during pregnancy), and it is very important to protect their mental as well as physical health [75]. This study fills a gap in the current literature by examining the associations between linkage to HIV care and postpartum depression among pregnant women in a rural setting in sub-Saharan Africa.

This study also adds to the current body of literature by examining the effects of linkage to care itself (Pre-ART care, which includes a package of services that may benefit mental health) versus the effects of linkage to care and being on ART. There has been little previous research attempting to tease apart the separate effects of ART medication and other components of HIV care on these outcomes. Linkage to HIV care and the opportunity to take advantage of other supportive services may result in lower stigma and/or depression, especially for pregnant women, who are particularly vulnerable to depression [14,22,23]. Although the current analyses are based on a relatively small sample size, the findings point to the possibility that ART is an important component of the effect of linkage to HIV care on postpartum depression.

Internalized HIV-related stigma and depression both include aspects such as self-blame and self-devaluation, and hence may be similar constructs. Although in this study we found these measures to be correlated, they appeared to be distinct aspects of the experiences of many newly diagnosed pregnant and postpartum women living with HIV in our sample. We found that internalized HIV-related stigma was a mediating factor in the association between linkage to HIV care and depression in this population.

The other variables that were found to be significantly related to postpartum depression in this setting are, for the most part, in agreement with existing literature. Intimate partner violence has consistently been shown to be related to adverse mental health outcomes in women [76], and has been shown to be an important predictor of perinatal depression [72]. Having an infant with health problems has also been found to be an important predictor in some settings [25,77,78]. Number of births was found to be an important predictor of postpartum depression in HIV-infected women in Zimbabwe [17], although in the opposite direction found in the current study. Our analyses also suggest important roles of family’s knowledge about the woman’s HIV-positive status and communication with the male partner in understanding postpartum depression for women living with HIV in this rural Kenyan setting. These same factors (with the exception of infant health status) also predicted HIV-related internalized stigma.

Our results should be evaluated in the context of the limitations of this study. Only 70% of the original sample could be located and participated in the follow-up assessment after the birth. However, as reported elsewhere, no important differences were detected between those women who could be located and interviewed postpartum and those lost-to-follow-up [63]. Although we hypothesized that lower internalized HIV-related stigma leads to lower postpartum depression in our mediation analyses, these variables were measured at the same time point (i.e., during the postpartum interview). Because pregnant women were interviewed at baseline before their initial ANC visit and before they knew their HIV status, we were not able to collect baseline data on internalized HIV-related stigma. In addition, a large proportion of women in the sample did not link to HIV care, meaning that we were not able to obtain data on their stage of HIV disease, and we only had self-report data on women’s current ART use.

Our findings are correlational and cannot provide definitive evidence for a causal role of linkage to HIV care in affecting depression or stigma. However, we have adjusted our analyses for socio-demographics and several key predictors from the literature. Ancillary analyses on a limited subsample of women included in the current analyses who completed an additional questionnaire during late pregnancy (n = 67) suggested that depression symptom severity was not very stable from late pregnancy to six weeks after the birth (Pearson *r* = 0.25). Thus, it is not likely that late pregnancy depression was a confounding variable in the association between linkage to care and postpartum depression. Repeating our main analysis on the limited subsample of women for whom we had late pregnancy data yielded a similar effect size for the association between linkage to care and postpartum depression controlling for depression at late pregnancy (*B* = −1.51, *p* = .07, n = 64 for the effect of linkage to care) compared to our main analyses where depression at late pregnancy was not controlled (*B* = −1.85, *p* = .04, N = 135; see Table 2).

Important strengths of the study include the prospective study design targeting a particularly vulnerable population: Participants were interviewed immediately before their first ANC visit and again around six weeks after the birth and only those participants who tested positive for HIV for the first time were included. Other strengths include the utilization of medical record data, rather than self-report, for our key predictor variable of linkage to care, and detailed analyses controlling for important variables that may be related to our outcome variables.

## Conclusions

Linkage to HIV care, especially when combined with ART, may predict lower postpartum depression symptoms (as well as lower postpartum depression rates) and lower internalized stigma among newly diagnosed postpartum women living with HIV. Our analyses suggest that linkage to care may potentially help prevent postpartum depression symptoms through helping women to cope with stigma and reducing internalized stigma. Thus, along with other health benefits, interventions focusing on linkage to care and initiation of ART may help to protect the mental health of pregnant and childbearing women in sub-Saharan Africa, with important benefits for maternal and child health.

## Abbreviations

PMTCT: Prevention of mother-to-child transmission
ART: Antiretroviral therapy
ANC: Antenatal care
EPDS: Edinburgh postpartum depression scale
IPV: Intimate partner violence
GEE: Generalized estimating equations
HASI-P: HIV/AIDS Stigma Instrument–PLWA
OR: Odds ratio

## References

[1] Leahy-Warren P, McCarthy G (2007). Postnatal depression: prevalence, mothers’ perspectives, and treatments. Arch Psychiatr Nurs.

[2] Chan SWC, Levy V, Chung TKH, Lee D (2002). A qualitative study of the experiences of a group of Hong Kong Chinese women diagnosed with postnatal depression. J Adv Nurs.

[3] Healey C, Morriss R, Henshaw C, Wadoo O, Sajjad A, Scholefield H, Kinderman P (2013). Self-harm in postpartum depression and referrals to a perinatal mental health team: an audit study. Arch Womens Ment Health.

[4] Murray L, Woolgar M, Cooper P, Hipwell A (2001). Cognitive vulnerability to depression in 5‐year‐old children of depressed mothers. J Child Psychol Psychiatry.

[5] Murray L, Cooper PJ (1996). The impact of postpartum depression on child development. Int Rev Psychiatr.

[6] Rahman A, Harrington R, Bunn J (2002). Can maternal depression increase infant risk of illness and growth impairment in developing countries?. Child Care Health Dev.

[7] Rahman A, Bunn J, Lovel H, Creed F (2007). Maternal depression increases infant risk of diarrhoeal illness:–a cohort study. Arch Dis Child.

[8] Rahman A, Iqbal Z, Bunn J, Lovel H, Harrington R (2004). Impact of maternal depression on infant nutritional status and illness: a cohort study. Arch Gen Psychiatry.

[9] Patel V, DeSouza N, Rodrigues M (2003). Postnatal depression and infant growth and development in low income countries: a cohort study from Goa, India. Arch Dis Child.

[10] O’Hara MW, Swain AM (1996). Rates and risk of postpartum depression-a meta-analysis. Int Rev Psychiatr.

[11] Parsons CE, Young KS, Rochat TJ, Kringelbach ML, Stein A (2012). Postnatal depression and its effects on child development: a review of evidence from low-and middle-income countries. Br Med Bull.

[12] Sawyer A, Ayers S, Smith H (2010). Pre-and postnatal psychological wellbeing in Africa: a systematic review. J Affect Disord.

[13] Villegas L, McKay K, Dennis CL, Ross LE (2011). Postpartum depression among rural women from developed and developing countries: a systematic review. J Rural Health.

[14] Peltzer K, Shikwane ME (2011). Prevalence of postnatal depression and associated factors among HIV-positive women in primary care in Nkangala district, South Africa. South Afr J HIV Med.

[15] Kapetanovic S, Christensen S, Karim R, Lin F, Mack WJ, Operskalski E, Frederick T, Spencer L, Stek A, Kramer F (2009). Correlates of perinatal depression in HIV-infected women. AIDS Patient Care STDS.

[16] Chibanda D, Mangezi W, Tshimanga M, Woelk G, Rusakaniko P, Stranix-Chibanda L, Midzi S, Maldonado Y, Shetty AK (2010). Validation of the Edinburgh postnatal depression scale among women in a high HIV prevalence area in urban Zimbabwe. Arch Womens Ment Health.

[17] Cyimana A, Andrews B, Ahmed Y, Vwalika B (2010). HIV/AIDS and postnatal depression at the University teaching Hospital, Lusaka, Zambia. Med J Zambia.

[18] Hartley C, Pretorius K, Mohamed A, Laughton B, Madhi S, Cotton MF, Steyn B, Seedat S (2010). Maternal postpartum depression and infant social withdrawal among human immunodeficiency virus (HIV) positive mother–infant dyads. Psychol Health Med.

[19] (UNAIDS) JUNPoHA (2013). 2013 Progress Report on the Global Plan Towards the Elimination of New HIV Infections among Children by 2015 and Keeping their Mothers Alive.

[20] Beaulière A, Touré S, Alexandre P-K, Koné K, Pouhé A, Kouadio B, Journy N, Son J, Ettiègne-Traoré V, Dabis F (2010). The financial burden of morbidity in HIV-infected adults on antiretroviral therapy in Cote d’Ivoire. PLoS ONE.

[21] Breman JG, Alilio MS, Mills A, Russell S (2004). The Economic Burden of Illness for Households in Developing Countries: a Review of Studies Focusing on Malaria, Tuberculosis, and Human Immunodeficiency Virus/Acquired Immunodeficiency Syndrome. Am J Trop Med Hyg.

[22] Taylor AL (2012). Social Support: A Predictor of Postpartum Depression among HIV-Positive and HIV-Negative Women.

[23] Bennetts A, Shaffer N, Manopaiboon C, Chaiyakul P, Siriwasin W, Mock P, Klumthanom K, Sorapipatana S, Yuvasevee C, Jalanchavanapate S (1999). Determinants of depression and HIV-related worry among HIV-positive women who have recently given birth, Bangkok, Thailand. Soc Sci Med.

[24] Kwalombota M (2002). The effect of pregnancy in HIV-infected women. AIDS Care.

[25] Dow A, Dube Q, Pence BW, Van Rie A (2014). Postpartum depression and HIV infection among women in Malawi. J Acquir Immune Defic Syndr.

[26] Turan J, Nyblade L (2013). HIV-related stigma as a barrier to achievement of Global PMTCT and maternal health goals: a review of the evidence. AIDS Behav.

[27] Colvin CJ, Konopka S, Chalker JC, Jonas E, Albertini J, Amzel A, Fogg K (2014). A Systematic Review of Health System Barriers and Enablers for Antiretroviral Therapy (ART) for HIV-Infected Pregnant and Postpartum Women. PloS One.

[28] Turan JM, Hatcher AH, Medema-Wijnveen J, Onono M, Miller S, Bukusi EA, Turan B, Cohen CR (2012). The role of HIV-related stigma in utilization of skilled childbirth services in rural Kenya: a prospective mixed-methods study. PLoS Med.

[29] Rahangdale L, Banandur P, Sreenivas A, Turan JM, Washington R, Cohen CR (2010). Stigma as experienced by women accessing prevention of parent-to-child transmission of HIV services in Karnataka, India. AIDS Care.

[30] Turan JM, Bukusi EA, Onono M, Holzemer WL, Miller S, Cohen CR (2011). HIV/AIDS stigma and refusal of HIV testing among pregnant women in rural Kenya: results from the MAMAS study. AIDS Behav.

[31] Wingood GM, Reddy P, Peterson SH, DiClemente RJ, Nogoduka C, Braxton N, Mbewu AD (2008). HIV stigma and mental health status among women living with HIV in the Western Cape, South Africa. S Afr J Sci.

[32] Thomas BE, Rehman F, Suryanarayanan D, Josephine K, Dilip M, Dorairaj VS, Swaminathan S (2005). How stigmatizing is stigma in the life of people living with HIV: a study on HIV positive individuals from Chennai, South India. AIDS Care.

[33] Akena D, Joska J, Obuku EA, Amos T, Musisi S, Stein DJ (2012). Comparing the accuracy of brief versus long depression screening instruments which have been validated in low and middle income countries: a systematic review. BMC Psychiatry.

[34] Onyebuchi-Iwudibia O, Brown A (2013). HIV and depression in Eastern Nigeria: The role of HIV-related stigma. AIDS Care.

[35] Charles B, Jeyaseelan L, Pandian A, Sam A, Thenmozhi M, Jayaseelan V (2012). Association between stigma, depression and quality of life of people living with HIV/AIDS (PLHA) in South India–a community based cross sectional study. BMC Public Health.

[36] Gupta R, Dandu M, Packel L, Rutherford G, Leiter K, Phaladze N, Percy-de Korte F, Iacopino V, Weiser SD (2010). Depression and HIV in Botswana: a population-based study on gender-specific socioeconomic and behavioral correlates. PLoS One.

[37] Steward WT, Chandy S, Singh G, Panicker ST, Osmand TA, Heylen E, Ekstrand ML (2011). Depression is not an inevitable outcome of disclosure avoidance: HIV stigma and mental health in a cohort of HIV-infected individuals from Southern India. Psychol Health Med.

[38] Li L, Lee S-J, Thammawijaya P, Jiraphongsa C, Rotheram-Borus MJ (2009). Stigma, social support, and depression among people living with HIV in Thailand. AIDS Care.

[39] Simbayi LC, Kalichman S, Strebel A, Cloete A, Henda N, Mqeketo A (2007). Internalized stigma, discrimination, and depression among men and women living with HIV/AIDS in Cape Town, South Africa. Soc Sci Med.

[40] Hatzenbuehler ML, O’Cleirigh C, Mayer KH, Mimiaga MJ, Safren SA (2011). Prospective associations between HIV-related stigma, transmission risk behaviors, and adverse mental health outcomes in men who have sex with men. Ann Behav Med.

[41] Tsai AC, Bangsberg DR, Frongillo EA, Hunt PW, Muzoora C, Martin JN, Weiser SD (2012). Food insecurity, depression and the modifying role of social support among people living with HIV/AIDS in rural Uganda. Soc Sci Med.

[42] Wagner GJ, Goggin K, Remien RH, Rosen MI, Simoni J, Bangsberg DR, Liu H (2011). A closer look at depression and its relationship to HIV antiretroviral adherence. Ann Behav Med.

[43] Weiss MG, Ramakrishna J (2006). Stigma interventions and research for international health. Lancet.

[44] Tsai AC, Bangsberg DR, Bwana M, Haberer JE, Frongillo EA, Muzoora C, Kumbakumba E, Hunt PW, Martin JN, Weiser SD (2013). How does antiretroviral treatment attenuate the stigma of HIV? Evidence from a cohort study in rural Uganda. AIDS Behav.

[45] Beard J, Feeley F, Rosen S (2009). Economic and quality of life outcomes of antiretroviral therapy for HIV/AIDS in developing countries: a systematic literature review. AIDS Care.

[46] Peterson K, Togun T, Klis S, Menten J, Colebunders R (2012). Depression and posttraumatic stress disorder among HIV-infected Gambians on antiretroviral therapy. AIDS Patient Care STDS.

[47] Patel R, Kassaye S, Gore-Felton C, Wyshak G, Kadzirange G, Woelk G, Katzenstein D (2009). Quality of life, psychosocial health, and antiretroviral therapy among HIV-positive women in Zimbabwe. AIDS Care.

[48] Wagner GJ, Ghosh-Dastidar B, Garnett J, Kityo C, Mugyenyi P (2012). Impact of HIV antiretroviral therapy on depression and mental health among clients with HIV in Uganda. Psychosom Med.

[49] Okeke EN, Wagner GJ (2013). AIDS treatment and mental health: evidence from Uganda. Soc Sci Med.

[50] Gilbert L, Walker L (2010). ‘My biggest fear was that people would reject me once they knew my status…’: stigma as experienced by patients in an HIV/AIDS clinic in Johannesburg, South Africa. Health Soc Care Community.

[51] Zuch M, Lurie M (2012). ‘A virus and nothing else’: the effect of ART on HIV-related stigma in rural South Africa. AIDS Behav.

[52] Gilbert L, Walker L (2009). “They (ARVs) are my life, without them I’m nothing”—experiences of patients attending a HIV/AIDS clinic in Johannesburg, South Africa. Health Place.

[53] Campbell C, Skovdal M, Madanhire C, Mugurungi O, Gregson S, Nyamukapa C (2011). “We, the AIDS people…”: how antiretroviral therapy enables Zimbabweans living with HIV/AIDS to cope with stigma. Am J Public Health.

[54] Bor J, Tanser F, Newell M, Barninghausen T (2012). In a study of a population cohort in South Africa, HIV patients on antiretrovirals had nearly full recovery of employment. Health Aff (Millwood).

[55] Thirumurthy H, Goldstein M, Zivin J (2008). The economic impact of AIDS treatment: labor supply in western Kenya. J Hum Resour.

[56] Tsai AC, Bangsberg DR, Weiser SD (2013). Harnessing poverty alleviation to reduce the stigma of HIV in Sub-Saharan Africa. PLoS Med.

[57] Makoae LN, Portillo CJ, Uys LR, Dlamini PS, Greeff M, Chirwa M, Kohi TW, Naidoo J, Mullan J, Wantland D (2009). The impact of taking or not taking ARVs on HIV stigma as reported by persons living with HIV infection in five African countries. AIDS Care.

[58] Pearson CR, Micek MA, Pfeiffer J, Montoya P, Matediane E, Jonasse T, Cunguara A, Rao D, Gloyd SS (2009). One year after ART initiation: psychosocial factors associated with stigma among HIV-positive Mozambicans. AIDS Behav.

[59] Roura M, Urassa M, Busza J, Mbata D, Wringe A, Zaba B (2009). Scaling up stigma? The effects of antiretroviral roll-out on stigma and HIV testing. Early evidence from rural Tanzania. Sex Transm Infect.

[60] Roura M, Wringe A, Busza J, Nhandi B, Mbata D, Zaba B, Urassa M (2009). Just like fever”: a qualitative study on the impact of antiretroviral provision on the normalisation of HIV in rural Tanzania and its implications for prevention. BMC Int Health Hum Rights.

[61] Thyssen A, Lange JH, Thyssen E, Reddi A (2013). Toward an AIDS-free generation with Option B+: reconceptualizing and integrating prevention of Mother to Child Transmission (PMTCT) with pediatric antiretroviral therapy initiatives. J Acquir Immune Defic Syndr.

[62] National AIDS and STI Control Program (2010). Sentinel Surveillance for HIV and Syphilis among Pregnant Women, 2010.

[63] Cuca YP, Onono M, Bukusi E, Turan JM (2012). Factors associated with pregnant women’s anticipations and experiences of HIV-related stigma in rural Kenya. AIDS Care.

[64] James J (2009). Sharing mechanisms for information technology in developing countries, social capital and quality of life. Soc Indic Res.

[65] Holzemer WL, Uys LR, Chirwa ML, Greeff M, Makoae LN, Kohi TW, Dlamini PS, Stewart AL, Mullan J, Phetlhu RD, Wantland D, Durrheim K (2007). Validation of the HIV/AIDS stigma instrument—PLWA (HASI-P). AIDS Care.

[66] Cox JL, Holden JM, Sagovsky R (1987). Detection of postnatal depression. Development of the 10-item Edinburgh Postnatal Depression Scale. Br J Psychiatry.

[67] Pop VJ, Komproe IH, Van Son MJ (1992). Characteristics of the Edinburgh post natal depression scale in The Netherlands. J Affect Disord.

[68] Zubaran C, Schumacher M, Roxo MR, Foresti K (2010). Screening tools for postpartum depression: validity and cultural dimensions. Afr J Psychiatry (Johannesbg).

[69] Tsai AC, Scott JA, Hung KJ, Zhu JQ, Matthews LT, Psaros C, Tomlinson M (2013). Reliability and validity of instruments for assessing perinatal depression in African settings: systematic review and meta-analysis. PLoS ONE.

[70] Halbreich U, Karkun S (2006). Cross-cultural and social diversity of prevalence of postpartum depression and depressive symptoms. J Affect Disord.

[71] Matthey S, Henshaw C, Elliott S, Barnett B (2006). Variability in use of cut-off scores and formats on the Edinburgh Postnatal Depression Scale–implications for clinical and research practice. Arch Womens Ment Health.

[72] Lancaster CA, Gold KJ, Flynn HA, Yoo H, Marcus SM, Davis MM (2010). Risk factors for depressive symptoms during pregnancy: a systematic review. Am J Obstet Gynecol.

[73] Krull JL, MacKinnon DP (2001). Multilevel modeling of individual and group level mediated effects. Multivar Behav Res.

[74] Hirnschall G, Doherty M, Shaffer N (2013). Is Option B+ the best choice?. Lancet.

[75] Psaros C, Geller PA, Aaron E (2009). The importance of identifying and treating depression in HIV infected, pregnant women: a review. J Psychosom Obstet Gynaecol.

[76] Romito P, Molzan Turan J, De Marchi M (2005). The impact of current and past interpersonal violence on women’s mental health. Soc Sci Med.

[77] Romito P, Turan JM, Neilands T, Lucchetta C, Pomicino L, Scrimin F (2009). Violence and women’s psychological distress after birth: an exploratory study in Italy. Health Care Women Int.

[78] Stewart RC, Bunn J, Vokhiwa M, Umar E, Kauye F, Fitzgerald M, Tomenson B, Rahman A, Creed F (2010). Common mental disorder and associated factors amongst women with young infants in rural Malawi. Soc Psychiatry Psychiatr Epidemiol.

